# Operations Factors Associated with Emergency Department Length of Stay: Analysis of a National Operations Database

**DOI:** 10.5811/westjem.2022.10.56609

**Published:** 2023-01-31

**Authors:** Maureen Canellas, Sean Michael, Kevin Kotkowski, Martin Reznek

**Affiliations:** * University of Massachusetts Medical School, Department of Emergency Medicine, Worcester, Massachusetts; † University of Colorado School of Medicine, Department of Emergency Medicine, Aurora, Colorado

## Abstract

**Introduction:**

Prolonged emergency department (ED) length of stay (LOS) has been shown to adversely affect patient care. We sought to determine factors associated with ED LOS via analysis of a large, national, ED operations database.

**Methods:**

We performed retrospective, multivariable, linear regression modeling using the 2019 Emergency Department Benchmarking Alliance survey results to identify associated factors of ED LOS for admitted and discharged patients.

**Results:**

A total of 1,052 general and adult-only EDs responded to the survey. Median annual volume was 40,946. The median admit and discharge LOS were 289 minutes and 147 minutes, respectively. R-squared values for the admit and discharge models were 0.63 and 0.56 with out-of-sample R-squared values of 0.54 and 0.59, respectively. Both admit and discharge LOS were associated with academic designation, trauma level designation, annual volume, proportion of ED arrivals occurring via emergency medical services, median boarding, and use of a fast track. Additionally, admit LOS was associated with transfer-out percentage, and discharge LOS was associated with percentage of high Current Procedural Terminology, percentage of patients <18 years old, use of radiographs and computed tomography, and use of an intake physician.

**Conclusion:**

Models derived from a large, nationally representative cohort identified diverse associated factors of ED length of stay, several of which were not previously reported. Dominant within the LOS modeling were patient population characteristics and other factors extrinsic to ED operations, including boarding of admitted patients, which was associated with both admitted and discharged LOS. The results of the modeling have significant implications for ED process improvement and appropriate benchmarking.

## INTRODUCTION

Emergency department (ED) length of stay (LOS) impacts a number of key patient-centered outcomes. Specifically, prolonged ED LOS adversely affects mortality,^1^–^3^ left without being seen rates,^1^,^4^ overall hospital LOS,^1^,^5^,^6^ and patient satisfaction.^7^,^8^ A small number of investigations describing causes for prolonged ED LOS exist. However, the body of literature is somewhat limited by the methodological approaches of the individual investigations, which generally have been characterized by small sample sizes, single-site studies or before-and-after study designs, which did not measure for all potential confounding factors thought or known to affect ED LOS. Nonetheless, the available literature in aggregate does suggest that the cause of prolonged ED LOS is multifactorial with potential contributing factors including patient population characteristics^9^–^12^ (eg, annual patient encounters, proportion of pediatric patients), intrinsic ED operations characteristics^13^–^15^ (eg, utilization of a low-acuity patient fast track), and extrinsic flow constraints^10^ (eg, hospital occupancy, elective surgical admissions). The relative paucity of investigations in this area of study coupled with the methodological limitations has precluded generalizability of conclusions; so there remains opportunity to improve our overall understanding of the constellation of factors contributing to ED LOS.

Despite demonstration of the deleterious effects of prolonged ED LOS and the identification of some potential causes, there has been little progress in improving ED LOS nationally over the past decades.^16^,^17^ Our study builds upon prior research by considering a diverse array of operational variables from greater than 1,000 EDs across the United States, including patient-population factors and intrinsic and extrinsic operational factors, allowing for a more robust and generalizable understanding of ED LOS-associated factors. Better identification of associated factor variables stands to inform efforts to improve ED patient flow and mitigate harms associated with prolonged LOS.

The purpose of our investigation was to determine factors associated with ED LOS using a large, national, ED operations database.

## METHODS

We used the 2019 Emergency Department Benchmarking Alliance (EDBA) survey results for general and adult-only EDs. The EDBA survey responses from pediatric EDs, specialty EDs (smaller EDs at specialty hospitals focused on specific specialties such as orthopedics or obstetrics and gynecology), and free-standing EDs were excluded from our investigation as they were not necessarily representative of the operational experiences of most EDs and were limited in number. The University of Massachusetts Medical School Institutional Review Board approved the investigation as exempt.

The EDBA is a not-for-profit, national consortium created to support and improve clinical operations of EDs. Among other efforts, the EDBA administers an annual ED operations survey to its member and past-member institutions. Within the survey instrument are several operations-related questions. Survey participation is voluntary. However, receiving de-identified results and aggregate analyses of the survey is a benefit afforded to participants and a primary motivator for membership in the consortium overall. The EDBA membership and survey development details are available in previous publications^18^ and at the EDBA website.^16^ We analyzed the EDBA survey administered and reported in 2019, which reflected reported ED data from January 1–December 31, 2018. A list of survey-related variable definitions can be found in the EDBA dictionary.^19^

We evaluated two primary outcomes: 1) ED LOS for patients admitted to an inpatient setting from the ED; and 2) ED LOS for patients discharged from the ED. While there may have been overlap in the potential factors affecting LOS for these two populations, the factors were anticipated to have affected the two groups differently; therefore, we analyzed the two outcomes separately. Separately considering these two populations was consistent with oversight entities such as the Centers for Medicare and Medicaid Services, which report ED LOS data for both patient populations.^20^

**Population Health Research Capsule**

What do we already know about this issue?

*Prolonged emergency department (ED) length of stay (LOS) has been shown to adversely affect patient care and staff satisfaction*.

What was the research question?

*Based on a national ED operations database, what factors are most associated with admit and discharge ED LOS?*

What was the major finding of the study?

*Median boarding time was a dominant variable for both admit (0.9, P<0.001) and discharge (0.18, P<0.001) LOS*.

How does this improve population health?

*These models may better guide managers when implementing initiatives to improve admit and discharge ED length of stay*.

We reviewed the 2019 EDBA survey instrument and identified candidate variables with face validity for potential associated factors of ED LOS. The following continuous and categorical variables were identified as candidates to be included in our subsequent analysis: academic (designation); trauma level (designation); ED volume (ie, annual patient encounters); percentage of high Current Procedural Terminology coding (CPT); percentage of patients under 18; admit percentage; hospital admit percentage from the ED (ie, percentage of all hospital admissions originating from the ED); transfer-out percentage (ie, percentage of ED patients transferred to another hospital); proportion of ED arrivals occurring via emergency medical services (EMS); median boarding (time); use of an intake or triage physician; use of an intake or triage advanced practice provider; use of a fast track; and the number per 100 patients for diagnostic studies that included electrocardiograms, radiographs, computed tomography (CT), magnetic resonance imaging (MRI), and ultrasounds. Based on prior reports, we perceived a potential for multicollinearity for trauma level designation with annual volume and academic status.^21^

However, we also anticipated that the referral patterns associated with being a Level I trauma center were likely to be an associated factor of ED flow independent of academic designation or annual volume.^22^,^23^ Therefore, we included Level I trauma center vs other as a candidate variable in modeling. Percentage of hospital admissions originating from the ED and MRIs per 100 patients were not reported by 51.5% and 44.6% of the institutions responding to the survey, respectively. We excluded these two candidate variables from final analysis for two reasons. First, their missingness percentages were high outliers compared to the other variables, which ranged from 0.6 to 26.7% with a median of 11.3% (Supplemental Table 1). In addition, the missing data was primarily from rural, non-academic, small-volume EDs, which would likely have led to significant bias if we imputed their data using dissimilar sites for their new values. In terms of variable values, there were no significant outliers identified for all included variables.

Subsequently, we created two separate multivariable, linear regression models predicting ED LOS for admitted patients and ED LOS for discharged patients. A random 70–30 split was used to construct training and validation sets. We applied a nonparametric missing value imputation algorithm using random forest, missForest,^24^ across the 16 independent variables with missing values within each set to allow for a more robust imputation. The algorithm assumed pairwise independence between observations but notably did not assume data being missing at random. Median and mean values did not appreciably differ between the original and imputed dataset (Supplemental Table 2). Of note, we chose missForest for its ability to impute across mixed-type data and the lack of studies clearly identifying another imputation technique as superior.

Variance inflation factors ranged from 1 to 3, indicating non-significant levels of multicollinearity^25^ (Table 1). With regard to our assumptions related to trauma level designation, the variance inflation factor was 1.92 for the admit model and 1.70 for the discharge model.

We conducted model validation by computing adjusted R-squared and out-of-sample R-squared values and used an alpha value of 0.05. All analyses were performed using R version 4.0.2 (R Foundation for Statistical Computing, Vienna, Austria).

## RESULTS

A total of 1,389 total EDs were surveyed by the EDBA with 1,335 responding (96% response rate). Of the responding EDs, we excluded 283 pediatric-only, specialty, and freestanding EDs, resulting in 1,052 EDs included in the analysis. The demographics for the included ED sites are reported in Table 2. The median annual patient volume was 40,946. The median admit and discharge LOS were 289 minutes (interquartile range [IQR] 122–184 minutes) and 147 minutes (IQR 237–359 minutes), respectively. Academic designation, trauma Level I designation, annual volume, transfer-out percentage, EMS arrival percentage, median boarding, and use of a fast track were significant associated factors of admit LOS (Supplemental Table 3).

Significant associated factors of discharge LOS were academic designation, trauma Level I designation, annual volume, high CPT percentage, percentage of patients <18 years old, EMS arrival percentage, median boarding, radiographs per 100 patients, CT per 100 patients, use of an intake or triage physician, and use of a fast track (Supplemental Table 4).

## DISCUSSION

Our regression modeling of the results of the 2019 EDBA survey, which included approximately one quarter of all EDs in the US, identified multiple factors associated with ED LOS that have not been previously reported. In addition, our results corroborated findings reported in prior investigations with more limited study populations. Overall, our results confirmed that factors associated with ED LOS are diverse and span three general categories: intrinsic ED operational factors; extrinsic operational factors; and the characteristics of the patient population served. Moreover, our investigation revealed that patient population characteristics and operational factors extrinsic to the ED dominated the associated factors associated with LOS for both admitted and discharged patients. The associated factors for admit and discharge LOS overlapped for the most part, highlighting that LOS for the two groups likely was influenced by common factors, although there were some differences worthy of consideration.

In general, the factors associated with admit LOS appeared to be dominated by characteristics related to the patient population served. Academic and trauma level designation have been reported previously as likely surrogates for acuity and complexity of patient populations.^18^ Furthermore, a high proportion of arrivals by EMS also has been associated with higher complexity of patient populations and higher admission rates in prior studies.^18^,^26^ These factors were all associated with increased admit LOS in our study. Additionally, annual patient volume was associated with longer admit LOS in our investigation. While larger centers likely care for more complex patient populations as they often serve as referral centers for specialty care, patient volume remained an independent associated factor of LOS.

Other factors associated with admit LOS included boarding time, transfer-out percentage, and presence of a fast track. Assuming that the transfer-out percentage primarily reflected lack of availability of specialized resources within the greater hospital, only one factor associated with admit LOS was intrinsic to ED operations: presence of a fast track. It is interesting to note that an operational strategy intended to focus on low-acuity patients (presumably more likely to be discharged) was associated with reduced LOS for admitted patients, likely confirming that the efficiencies from split flow described in a limited set of academic EDs hold true more broadly.^14^ While the EDBA survey data did not allow for causal investigation, this finding highlighted that ED operational processes are complex and intertwined. In aggregate, our study results revealed that admit LOS is predominantly associated with factors outside ED operations.

Factors associated with discharge LOS included all the associated factors of admit LOS with the exception of transfer-out percentage. Given that the transfer-out percentage likely reflected available hospital resources, this association with admit LOS but not discharge LOS has face validity given that most discharged patients are less likely to require subspecialty expertise. In addition to the associated factors discussed above, for admit LOS, discharge LOS was associated with several additional variables. Greater proportion of higher CPT coding was associated with increased discharge LOS. Although it may be influenced by local documentation and coding/billing practices, the CPT coding system is designed to represent patient acuity and complexity. Patient age <18 also was a significant associated factor of discharge LOS, with a larger percentage of pediatric patients having been associated with shorter discharge LOS. This appears to be consistent with prior reports that pediatric ED patients tend to have lower acuity and complexity compared to their adult counterparts.^27^ It remains unclear why proportion of pediatric patients and higher CPT codes would be associated with discharge LOS but not admit LOS.

Also associated with discharge LOS, but not admit LOS, were utilization of plain film radiography and CT. It is intuitive that performing more CTs and radiographs could prolong LOS for all patients. However, it is interesting that imaging utilization was not associated with admit LOS. Intuitively, admitted patients would have been characterized by higher acuity and complexity than discharged patients and likely would have required these resources to a greater degree. Two possible explanations may be that 1) for admitted patients, the additional time for imaging did not affect their overall LOS because the time waiting for imaging ran in parallel with other factors influencing LOS, or 2) other factors such as boarding became so dominant for admitted patients that imaging no longer was significant within the multivariable analysis. We postulate that the imaging utilization variables likely represent both a surrogate for patient population, such as acuity, and internal ED operational factors, such as local practices and practice cultures related to performing more or fewer imaging studies.

The remaining associated factor of discharge LOS was the presence of an intake or triage physician. (The EDBA survey did not differentiate between those two different models and terms.) Interestingly, physician intake was associated with longer discharge LOS. We postulate that this seemingly paradoxical finding did not imply causality but rather implied that this operational strategy was being implemented predominantly in EDs already challenged in patient flow due to other factors. Whether the presence of a physician in triage is an effective flow intervention was not possible to determine from our study.

Our finding that boarding was associated with both admit and discharge LOS warrants further reflection. De facto, boarding is a component of admit LOS; so its association with admit LOS was not unexpected. However, the finding of boarding being associated with discharge LOS has broader implications. Emergency departmemt operations leaders anecdotally have reported being held accountable for ED LOS for discharged patients, rather than all or admitted patients, under the premise that the discharged patient LOS is entirely under ED operational control. While our study was not designed to determine causality, our findings appeared to refute this notion, as boarding (among other non-intrinsic ED factors) was associated with prolonged LOS for discharged patients. Perhaps more importantly, boarding differs from the other extrinsic factors uncovered in our investigation in that it is a relatively manageable contributor to ED LOS.^10^,^28^

In general, our results are consistent with prior reports related to factors associated with ED LOS. Prior studies also identified ED volume,^9^,^10^ EMS arrival percentage,^9^ boarding or crowding levels,^9^,^10^ and academic designation^18^ as associated factors of ED LOS. One prior study showed that dedicated pediatric EDs were characterized by shorter LOS for discharged patients when compared to adult EDs.^29^ Our results related to the proportion of pediatric patients may be consistent with this result; however, the prior investigation differed in its methodology in that it compared dedicated pediatric EDs to general and adult-only EDs. Therefore, the prior study results may have reflected operational processes rather than the patient population itself. Our results also are consistent with prior reports showing flow improvements due to implementation of a fast track.^13^,^30^ Finally, with regard to our findings that admission percentage was not associated with LOS, a prior investigation did report that ED LOS increased on days that the admit percentage was higher from the ED. The two studies differed significantly in methodology, and it appears that the prior study’s results more likely reflected flow constraints related to daily variability, which was not measured in the EDBA survey tool. Therefore, it appears that the two investigations’ findings are not necessarily contradictory.

Our findings have significant implications for ED flow improvement efforts. In addition to highlighting specific factors associated with ED LOS across a large proportion of the EDs in the US, our study results show clearly that patient population-related factors dominate the list of variables associated with ED LOS. This observation underscores prior reports that cite the importance of appropriate benchmarking of ED operational outcomes for the purposes of ED process improvement guidance.^18^,^21^ Comparing EDs with significantly different characteristics such as patient volumes, trauma level designation or academic vs non-academic EDs for the purposes of guiding operational management efforts may be ill advised in light of the results of our investigation that provide additional evidence that they are not likely to be relevant comparators.

In addition, our results highlight that the constellation of factors associated with admit LOS and discharge LOS overlap more than they differ, but they appeared to differ predominantly in factors that reflect intrinsic ED operational factors. This implies that when developing internal ED initiatives aimed at improving LOS, ED leadership should consider admitted and discharged patient populations separately when designing interventions and tracking metrics. Finally, our results provide additional evidence that reducing or eliminating boarding stands to be a pivotal ED flow strategy to reduce LOS for both admitted and discharged patients.

This was a retrospective, survey-based investigation. Overall, the survey exhibited a high response rate at 96%; however, there were more limited response rates for some individual survey questions. We employed validated methodology to impute missing data; however, missingness remains a potential limitation of our investigation. In particular, we excluded two candidate variables due to excessive missingness: percentage of hospital admissions originating from the ED; and MRIs per 100 patients. It is possible these factors may have been associated with ED LOS but remained unmeasured in our study design. The survey instrument was administered to EDBA members and past member EDs, rather than a random sampling of US EDs, which could have introduced sampling bias.

While reports of the total number of EDs and hospitals in the US in 2018 vary,^31^,^32^ it appears that the 1,389 EDs surveyed represented approximately a quarter of all US EDs at the time (likely even greater when excluding pediatric, specialty and free-standing EDs as was done in our methodology). While the lack of randomization must be considered when interpreting the results, the study population did represent a sizable proportion of general EDs. The EDBA survey instrument had been developed and refined over 25 years by experts in ED operations; nonetheless, the survey was not necessarily designed as a tool to include specifically all potential factors associated with ED LOS. Therefore, some factors may have been unmeasured by the survey instrument. Finally, responses to the survey were reported by participants as an annual aggregate value; so temporal factors potentially associated with ED LOS, such as daily or seasonal variability, could not be accounted for in our investigation. Data was reported at the level of the ED, rather than at the patient level; so caution is warranted in making inferences or predictions about an individual patient’s LOS, as our focus was on the overall performance of the ED as a whole.

## LIMITATIONS

As with any survey-based investigation, data integrity may have been limited by response bias, although the EDBA survey encompassed about a quarter of all EDs in the nation and remains the largest national ED metrics database. In addition, as with any survey-based investigation, we could not be certain of accurate and complete responses from survey participants. However, the EDBA survey instrument incorporated widely accepted and well-defined data definitions,^19^ lessening concerns related to accuracy.

Our analytical methodology also had limitations. Candidate variables were selected based on expert and author consensus of mechanistic plausibility. The ratio of potential associated factors to outcomes dictated that not all variables available from the EDBA be included in our models, and it is possible that excluded variables may have also been associated with ED LOS. In addition, our approach assumed linear relationships, and there remains a possibility of non-linear relationships among associated factors and with the outcomes. Because our goal was to describe factors associated with LOS at the level of the ED (to inform systems intervention opportunities, as opposed to predicting LOS for any given individual patient), we prioritized creating human-interpretable and more parsimonious models.

Constructing non-linear or “black box” machine-learning models would have been computationally feasible but impractical for our objective, as their interpretation is far less intuitive. Instead, we accepted the limitations of linearity assumptions to obtain the benefit of quantitative and intervenable model outputs (eg, a finding that “on average, having a fast track is associated with a 19-minute reduction in admitted patient LOS” is much more actionable than “presence of a fast track contributes to 6% of the variance in LOS”). Since our analysis occurred at the level of the ED, the other assumptions of linear regression were less limiting and easily verifiable, such as normality and homoscedasticity of the residuals.

We also noted a tendency for smaller, rural, non-trauma centers to contribute relatively more to data missingness. While the missing data was imputed with robust techniques, it remains unclear how this tendency may have affected the models. Lastly, our selected imputation algorithm, missForest, assumed pairwise independence between sites. Intuitively, we believed this to be a reasonable assumption for the majority of EDBA member institutions. However, some EDs in the database were part of multi-ED health systems, opening the possibility of them not being completely independent from other EDs within their health system.

## CONCLUSION

Models derived from a large, nationally representative cohort identified diverse associated factors of ED length of stay. Factors extrinsic to ED operations and patient population-associated factors were dominant within the modeling for both admit and discharge LOS. Notably, boarding of admitted patients was associated with not only admit LOS but also LOS for discharged patients, a subset of ED patients not directly subject to boarding. While the constellation of factors associated with admit LOS and discharge LOS predominantly overlapped, discharge LOS exhibited association with more factors intrinsic to ED operations than admit LOS. The results of our investigation have significant implications for appropriate benchmarking and ED process improvement efforts.

## Supplementary Information



## Figures and Tables

**Table 1:** Variance inflation factors for each variable across the admit and discharge models.

Variable	VIF for admit model variables	VIF for discharge model variables
Academic designation (vs not)	1.97	1.89
Trauma level 1 designation (vs not)	1.92	1.70
Annual volume (per patient)	2.23	2.32
Percentage of high Current Procedural Terminology coding	1.31	1.39
Percentage of patients <18 years old	1.63	1.64
Admit percentage	2.73	2.93
Transfer-out percentage	1.64	1.61
Emergency medical services arrival percentage	2.28	2.40
Median boarding time (in minutes)	1.43	1.45
Electrocardiograms per 100 patients	2.08	2.09
Radiographs per 100 patients	2.22	2.42
Computed tomography per 100 patients	2.37	2.51
Ultrasounds per 100 patients	1.33	1.53
Use of an intake physician (vs none)	1.42	1.46
Use of an intake advanced practice provider (vs none)	1.30	1.29
Use of a fast track (vs none)	1.54	1.55

*VIF*, variance inflation factor.

**Table 2:** Demographics of emergency departments included in dataset.

	Total (N)	Percent of total
Community type		
Suburban	464	44.1%
Urban	326	31.0%
Rural	260	24.7%
No response	2	0.2%
Academic designation		
Yes	248	23.6%
No	797	75.8%
No response	7	0.7%
Trauma level designation		
Level I	134	12.7%
Level II	132	12.5%
Level III	133	12.6%
Level IV	95	9.0%
Not a trauma center	552	52.5%
No response	6	0.6%
Annual encounters		
More than 120,000	12	1.1%
100,000–120,000	23	2.2%
80,000–100,000	70	6.7%
60,000–80,000	167	15.9%
40,000–60,000	263	25.0%
20,000–40,000	321	30.5%
Less than 20,000	190	18.1%
No response	6	0.6%
