# Supplementary material for: Operations Factors Associated with Emergency Department Length of Stay: Analysis of a National Operations Database
Source: West J Emerg Med. 2023 Jan 31;24(2):178–84. doi: 10.5811/westjem.2022.10.56609 (PMC10047726; doi:10.5811/westjem.2022.10.56609)
Supplement: Supplementary file 1 [file wjem-24-178-s001.docx]

**Supplemental Table 1.** Absolute and percent of missing values in original dataset for each variable. (LOS, length of stay)

| Variables | Frequency of Missing Values | Percentage of Missing Values (%) |
| --- | --- | --- |
| Electrocardiograms per 100 patients | 281 | 26.7 |
| Median boarding time (in min) | 276 | 26.2 |
| Ultrasounds per 100 patients | 261 | 24.8 |
| Use of an intake physician (vs. none) | 220 | 20.9 |
| Use of an intake advanced practice provider (vs. none) | 220 | 20.9 |
| Use of a fast track (vs. none) | 220 | 20.9 |
| X-rays per 100 patients | 211 | 20.1 |
| Computed tomography scans per 100 patients | 178 | 16.9 |
| Emergency medical services arrival percentage | 133 | 12.6 |
| Transfer out percentage | 105 | 10 |
| Percentage of patients under 18 years old | 75 | 7.1 |
| Percentage of high current procedural terminology coding | 59 | 5.6 |
| Admit LOS (in min) | 48 | 4.6 |
| Discharge LOS (in min) | 34 | 3.2 |
| Admit percentage | 17 | 1.6 |
| Academic designation (vs. not) | 7 | 0.7 |
| Trauma level 1 designation (vs. not) | 6 | 0.6 |
| Annual volume (per patient) | 6 | 0.6 |

**Supplemental Table 2.** Pre- and post-imputation medians and means for each variable and model.

| Variable | Original Dataset | | Admit Train Imputed Dataset | | Admit Test Imputed Dataset | | Discharge Train Imputed Dataset | | Discharge Test Imputed Dataset | |
| --- | --- | --- | --- | --- | --- | --- | --- | --- | --- | --- |
|  | Median | Mean | Median | Mean | Median | Mean | Median | Mean | Median | Mean |
| Percentage of high current procedural terminology coding | 0.73 | 0.71 | 0.73 | 0.71 | 0.72 | 0.71 | 0.73 | 0.71 | 0.72 | 0.71 |
| Percentage of patients under 18 years old | 0.13 | 0.12 | 0.12 | 0.12 | 0.13 | 0.13 | 0.12 | 0.12 | 0.13 | 0.13 |
| Admit percentage | 0.19 | 0.19 | 0.19 | 0.19 | 0.18 | 0.19 | 0.19 | 0.19 | 0.18 | 0.18 |
| Transfer out percentage | 0.02 | 0.03 | 0.02 | 0.03 | 0.02 | 0.03 | 0.02 | 0.03 | 0.02 | 0.03 |
| Emergency medical services arrival percentage | 0.18 | 0.18 | 0.18 | 0.19 | 0.17 | 0.18 | 0.18 | 0.19 | 0.18 | 0.18 |
| Median boarding time (in min) | 96.5 | 112.77 | 93.95 | 110.32 | 87.2 | 99.3 | 91 | 108.05 | 93.93 | 106.59 |
| Electrocardiograms per 100 patients | 29 | 29.92 | 30 | 30.36 | 28.19 | 29.6 | 30 | 30.65 | 29 | 29.07 |
| X-rays per 100 patients | 41 | 41.56 | 42 | 42.21 | 42.86 | 42.18 | 42 | 42.32 | 41 | 41.81 |
| Computed tomography scans per 100 patients | 23 | 24.76 | 23 | 24.71 | 25 | 26.4 | 24 | 25.05 | 23 | 25.18 |
| Ultrasounds per 100 patients | 6 | 6.93 | 6 | 6.87 | 6.16 | 6.87 | 6.11 | 6.92 | 6 | 6.83 |

**Supplemental Table 3.** Admit length of stay model variables and respective coefficients and significance.

| Variable | Regression Coefficient  (for length of stay in minutes) | P-value |
| --- | --- | --- |
| Intercept | 168.1 | <0.001 |
| Academic designation (vs. not) | 25.79 | <0.01 |
| Trauma level 1 designation (vs. not) | 27.71 | <0.01 |
| Annual volume (per patient) | 6.75e-4 | <0.001 |
| Percentage of high current procedural terminology coding | 7.66 | 0.76 |
| Percentage of patients under 18 years old | -79.90 | 0.13 |
| Admit percentage | 10.82 | 0.83 |
| Transfer out percentage | 320.6 | <0.05 |
| Emergency medical services arrival percentage | 107.8 | <0.05 |
| Median boarding time (in min) | 0.90 | <0.001 |
| Electrocardiograms per 100 patients | -4.86e-2 | 0.89 |
| X-rays per 100 patients | 5.47e-2 | 0.88 |
| Computed tomography scans per 100 patients | -0.46 | 0.21 |
| Ultrasounds per 100 patients | 0.24 | 0.69 |
| Use of an intake physician (vs. none) | 3.36 | 0.74 |
| Use of an intake advanced practice provider (vs. none) | 5.49 | 0.57 |
| Use of a fast track (vs. none) | -18.76 | <0.01 |

The model’s root mean squared error, mean absolute error, and mean absolute percentage error were 59.05, 41.96, and 0.14, respectively.

**Supplemental Table 4.** Discharge length of stay model variables and respective coefficients and significance.

| Variable | Regression Coefficient  (for length of stay in minutes) | P-value |
| --- | --- | --- |
| Intercept | 62.33 | <0.001 |
| Academic designation (vs. not) | 9.32 | <0.05 |
| Trauma level 1 designation (vs. not) | 26.81 | <0.001 |
| Annual volume (per patient) | 3.43e-4 | <0.001 |
| Percentage of high current procedural terminology coding | 49.93 | <0.001 |
| Percentage of patients under 18 years old | -111.5 | <0.001 |
| Admit percentage | 0.14 | 0.99 |
| Transfer out percentage | 40.64 | 0.60 |
| Emergency medical services arrival percentage | 87.55 | <0.001 |
| Median boarding time (in min) | 0.18 | <0.001 |
| Electrocardiograms per 100 patients | -0.18 | 0.32 |
| X-rays per 100 patients | 0.41 | <0.05 |
| Computed tomography scans per 100 patients | 0.42 | <0.05 |
| Ultrasounds per 100 patients | -0.50 | 0.21 |
| Use of an intake physician (vs. none) | 16.35 | <0.01 |
| Use of an intake advanced practice provider (vs. none) | 5.80 | 0.26 |
| Use of a fast track (vs. none) | -8.07 | <0.05 |

The model’s root mean squared error, mean absolute error, and mean absolute percentage error were 28.80, 20.99, and 0.14, respectively.
